# Forging resilient warriors within: exercise’s epic role in training innate immunity and taming inflammation’s storm

**DOI:** 10.3389/fimmu.2026.1777470

**Published:** 2026-04-29

**Authors:** Yahui Li, Dongdong Wang

**Affiliations:** College of Physical Education and Health Science, Yibin University, Yibin, Sichuan, China

**Keywords:** epigenetic reprogramming, exercise immunology, inflammation resolution, sustainable development, trained immunity

## Abstract

The concept of “trained immunity,” which refers to a type of long-term immunological memory within innate cells, has significantly challenged the traditional division between the non-specific innate and antigen-specific adaptive immune systems. Simultaneously, it is now understood that the resolution of inflammation is not just a passive return to homeostasis but rather an active, strictly regulated biological process. This comprehensive review synthesizes these evolving concepts by hypothesizing that exercise, through its pleiotropic effects on cellular metabolism, may strongly stimulate both the active resolution of inflammation and the formation of innate immunological memory via epigenetic control (emerging evidence from non-exercise models like Bacillus Calmette-Guérin (BCG) vaccination and β-glucan exposure suggests parallels, but direct causation in exercise remains associative). The review describes the biological foundations of this relationship, including the metabolic changes that define trained immunity and the epigenetic reprogramming of myeloid progenitors. It explores the central role of myokines, particularly Interleukin-6 (IL-6), which act as critical mediators, steering the immune response toward a pro-resolving phenotype, while acknowledging contributions from other factors such as IL-15, catecholamines, and neural signals. Furthermore, the review outlines how exercise promotes the synthesis of specialized pro-resolving mediators (SPMs) and enhances efferocytosis, a key cellular mechanism for restoring tissue homeostasis. By providing a unifying framework, this analysis offers a mechanistic explanation for the profound health benefits of exercise, from enhanced immunosurveillance to the prevention of chronic inflammatory diseases. The review concludes by highlighting significant knowledge gaps and advocating for the use of multi-omics and *in vivo* models to fully elucidate this complex nexus and translate these discoveries into novel therapeutic strategies.

## Introduction

The traditional understanding of the immune system is based on a clear division of labor: the adaptive branch creates a slower, antigen-specific memory response, whereas the innate branch offers a quick, non-specific first line of defense (1–4). After an initial stimulus, innate immune cells such as monocytes, macrophages, dendritic cells, neutrophils, natural killer (NK) cells, and tissue-resident macrophages undergo long-term functional reprogramming, allowing them to respond more strongly to a subsequent, unrelated assault (1, 5, 6). The physiological mechanisms that drive this enhanced responsiveness are multifaceted, involving a complex interplay between epigenetic and metabolic changes (1, 7, 8). Importantly, trained immunity is induced in the bone marrow, where long-lived progenitors like hematopoietic stem cells (HSCs) are reprogrammed to propagate the trained phenotype to progeny cells. At the same time, it is becoming more well recognized that inflammation resolution is a very active process as opposed to a passive one (9). Instead of simply dissipating, inflammation is actively curtailed and tissue homeostasis restored by a distinct class of molecules known as SPMs (9–11). These lipid mediators, derived from essential fatty acids, not only dampen the inflammatory cascade but also actively promote host defense and the clearance of pathogens and cellular debris (9, 12, 13). The dual nature of these processes—both anti-inflammatory and pro-host defense—distinguishes them from classical immunosuppression (12).

The relationship between exercise and immune function mirrors this complexity, defying a simple linear model (14). Early research focused on basic changes in immune cell counts (15, 16). By the 1980s, a more complex understanding had developed, represented by the “J-shaped” curve, which postulates that while prolonged, intense training can increase the incidence of minor infections, especially of the upper respiratory tract, moderate exercise lowers the risk of infection when compared to a sedentary lifestyle (15, 17, 18). However, there is still much disagreement on the “open window” theory, which postulates a brief period of immunosuppression following intense exercise. However, there is evidence that suggests acute exercise may actually improve immune surveillance (16, 19, 20). This review seeks to unify these seemingly disparate fields by proposing a hypothesis-driven mechanistic framework in which exercise is posited to serve as a potent physiological modulator of innate immunity (21, 22). The main argument is that exercise actively promotes the resolution of inflammation and the development of trained immunity (23, 24). Evidence suggests how exercise-induced signals, particularly myokines released from contracting skeletal muscle, orchestrate the epigenetic and metabolic reprogramming of innate immune cells (25–27). Concurrently, Exercise increases vital cellular functions including efferocytosis and encourages the production of pro-resolving lipid mediators (28, 29). This dual-pronged action provides a comprehensive explanation for the pleiotropic health benefits of exercise, from enhanced pathogen defense to the prevention of chronic inflammatory diseases (28, 30, 31). By dissecting these molecular and cellular pathways, this review aims to establish exercise as a powerful, non-pharmacological tool for immunomodulation.

### The foundational function of innate immune memory

The long-held belief that memory is the sole realm of the adaptive immune system is being challenged by the idea of trained immunity, which represents a fundamental shift in immunological theory (32). This section provides a detailed exposition of innate immune memory, its molecular basis, and its critical distinction from adaptive immunity.

### Defining trained immunity and its mechanisms

Long-term, non-specific improvement of innate immune cell function is a hallmark of trained immunity, allowing for a stronger reaction to later, frequently unrelated immunological stimuli (1, 33, 34). This stands in stark contrast to adaptive immunity, which is mediated by T and B cells and is extremely specific to a given antigen (1, 35, 36). A wide variety of stimuli, such as endogenous sterile stimuli like oxLDL and microbial ligands like β-glucan and BCG vaccine, can cause the induction of trained immunity (1, 37).

How this memory endures in innate immune cells with comparatively short lifespans, including neutrophils and monocytes, is a key conundrum of trained immunity (1, 38, 39). Reprogramming long-lived myeloid progenitor cells is the solution to this dilemma. The initial training stimulus leaves an immunological mark on bone marrow-derived HSCs in addition to influencing mature, circulating immune cells (1, 40, 41). These reprogrammed HSCs then generate functionally adapted progeny cells that maintain the “trained” phenotype for months to years after the initial exposure (1, 41, 42). This fundamental mechanism explains how the clinical effects of an intervention, such as BCG vaccination, can last for at least five years, as seen in studies on children in Uganda (1, 43, 44). This suggests that for exercise to induce lasting trained immunity, it must also influence the intricate regulatory network of HSCs, a significant area for future investigation [emerging evidence from animal models shows exercise affects HSC proliferation and lineage commitment, but direct links to trained immunity reprogramming remain associative and require further validation (45, 46)]. Furthermore, the establishment of durable trained immunity requires precise negative regulation to prevent pathological overactivation. TRIM29 (tripartite motif-containing protein 29), an E3 ubiquitin ligase, serves as a key checkpoint that simultaneously enables training of innate immunity and actively tames inflammation. TRIM29 is rapidly induced in alveolar macrophages and mucosal epithelial cells upon pathogen encounter and functions as a selective negative regulator of innate responses (47). It suppresses type-I interferon production, NF-κB activation, and proinflammatory cytokine release by directly ubiquitinating and targeting critical signaling adapters—such as STING (in DNA-sensing pathways) and MAVS (in RNA-sensing pathways)—for proteasomal degradation (48, 49). By dampening these pathways, TRIM29 prevents excessive inflammatory storms and tissue damage while still permitting the epigenetic and metabolic reprogramming that underlies long-term innate immune memory. This balanced “train-and-tame” action is particularly relevant in mucosal sites where repeated microbial exposure occurs, and it provides a molecular explanation for how physiological stressors (including exercise) can promote beneficial trained immunity without tipping into chronic inflammation. Animal models, particularly in mice, have demonstrated that exercise enhances HSC proliferation and function. For instance, endurance exercise mobilizes developmentally early stem cells into peripheral blood and increases their numbers in bone marrow, potentially improving tissue regeneration (50). Additionally, exercise training increases the percentage of HSCs in the vascular niche of bone marrow and promotes cell proliferation, which may contribute to sustained immune adaptations (51). In models of enhanced physical activity, such as transgenic mice with increased muscle activity, HSCs exhibit reduced aging-related decline, with improved pool size and functional capacity (52). These effects on HSC progeny could underpin exercise’s role in propagating trained immunity systemically, though direct epigenetic links in exercise contexts warrant further study.

### Epigenetic and metabolic reprogramming as core drivers

The functional changes that define trained immunity are underpinned by a profound intracellular rewiring of gene transcription and metabolism (1, 53, 54). These two pathways must interact to induce and maintain innate immunological memory (1, 55).

#### Epigenetic mechanisms

Epigenetic reprogramming serves as the master switch for trained immunity. These changes can endure through cell divisions and modify gene expression without altering the underlying DNA sequence (56, 57). Histone alterations, which control DNA’s accessibility to transcriptional machinery, are essential to this process (58). Specifically, trained immunity is characterized by the acquisition of permissive marks such as H3K27ac marks at distal enhancers, H3K4me3 marks at gene promoters and H3K4me1 at enhancers (1, 59–61). However, repressive marks like H3K9me2 and H3K9me3 also contribute by silencing non-inflammatory genes, ensuring a balanced chromatin landscape. These changes result in euchromatin, a more open chromatin structure that makes it easier for transcription factors and other regulatory proteins to access and control gene expression (57, 62, 63). Following additional stimulation, this leads to increased transcription of inflammatory genes like TNFα and IL6 (58, 64). Exercise can directly influence this epigenetic landscape in immune cells. For example, early-life regular exercise in mice induces immunometabolic epigenetic modifications, enhancing anti-inflammatory immunity in middle age via changes in histone acetylation and DNA methylation patterns that overlap with trained immunity signatures (65). In humans, resistance exercise alters epigenetic marks in leukocytes, affecting pathways related to energy metabolism and inflammation, potentially distinct from or synergistic with trained immunity (66). These findings provide a direct link between exercise and long-term epigenetic reprogramming, though more studies are needed to delineate exercise-specific effects.

#### Metabolic reprogramming (immunometabolism)

**Figure 1:**
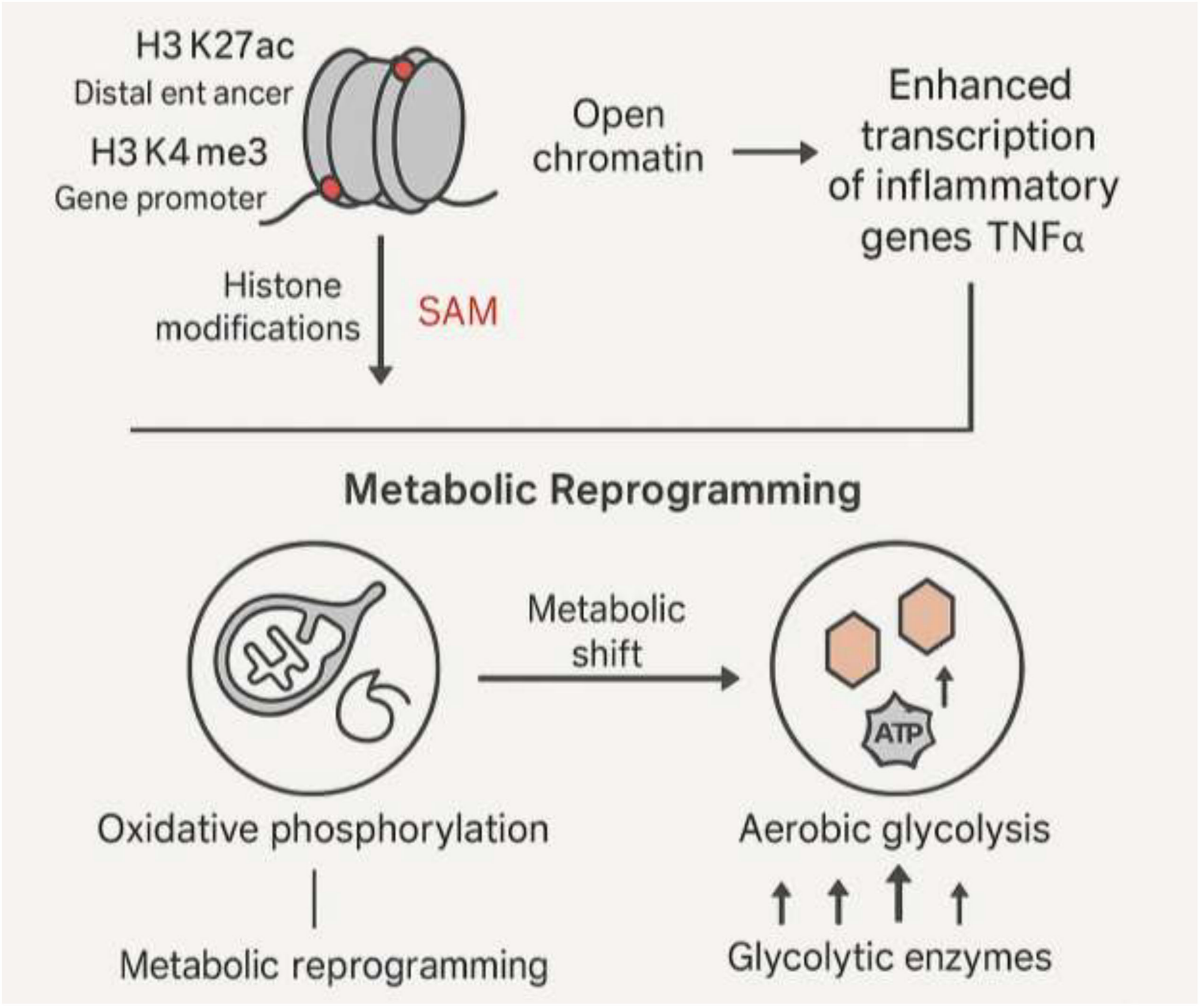
Epigenetic and metabolic reprogramming driving trained immunity.

The metabolic state of an immune cell is not a fixed characteristic; rather, it is a dynamic program that is modulated in response to immunological stimuli (67–69). While a shift from oxidative phosphorylation (OXPHOS) to aerobic glycolysis (often referred to as a Warburg-like effect) is associated with some aspects of trained immunity, this is not a universal feature. Instead, trained immunity involves increased activity in multiple metabolic pathways, including glycolysis, OXPHOS, the tricarboxylic acid (TCA) cycle, amino acid metabolism (e.g., glutaminolysis), and lipid metabolism (54). These diverse changes provide the energy and precursors needed for robust immune responses and epigenetic remodeling (67, 70). This metabolic shift is regulated by key metabolic sensors, especially HIF-1α (67, 71, 72). The activation of HIF-1α upregulates the expression of numerous glucose transporters and glycolytic enzymes, thereby increasing the overall rate of glycolysis (67, 73). This metabolic reprogramming is not merely a consequence of trained immunity but is a prerequisite for it (74). The central connection is that certain metabolites can directly affect the enzymes involved in epigenetic remodeling (75, 76). For example, S-adenosylmethionine (SAM) serves as a methyl donor for DNA methyltransferases, such as DNMT3A (77). This direct, metabolic-fueled link to epigenetic changes suggests a powerful causal pathway: any intervention, such as exercise, that alters cellular metabolism could potentially initiate or modulate trained immunity by providing the necessary metabolic substrates for these long-term epigenetic changes (78–80) (**Figure 1**).

Epigenetic modifications, including H3K27ac at enhancers and H3K4me3 at promoters, open chromatin and enhance transcription of inflammatory genes like TNFα. In parallel, metabolic reprogramming shifts immune cells from OXPHOS to aerobic glycolysis, regulated by metabolites like SAM and sensors like HIF-1α. These metabolic intermediates fuel epigenetic enzymes, linking metabolism to chromatin remodeling. Together, epigenetic and metabolic rewiring establish long-term innate immune memory, enabling heightened responses upon secondary stimulation.

## Skeletal muscle as a mediator: the myokine-immunometabolism axis

Skeletal muscle is linked with locomotion and metabolism for a long time but a more recent paradigm shift has established it as a key endocrine organ that communicates with various tissues throughout the body (25, 81, 82). The release of bioactive compounds called myokines mediates this communication (83). This section will explore how exercise-induced myokines act as critical intermediaries between physical activity and systemic immune modulation.

### Myokines: the endocrine arm of exercise

Small proteins and peptides known as myokines are produced and secreted in response to skeletal muscle contraction, especially during exercise (25, 84). Numerous physiological effects are mediated by these signaling molecules, including immunological response, metabolic process regulation, and whole-body homeostasis maintenance (25, 85). Prominent examples of myokines include Interleukin-6 (IL-6), Decorin, Myostatin, and Irisin (25, 86). These molecules facilitate crucial inter-organ crosstalk, influencing distant tissues like adipose tissue, the liver, and the brain (25).

### Interleukin-6: a pleiotropic hub

Interleukin-6 is arguably the most extensively studied myokine, and its role in immune regulation is highly complex and nuanced (87, 88). Despite being a well-known pro-inflammatory cytokine, exercise-induced IL-6 is frequently said to have strong anti-inflammatory properties (15). This apparent contradiction highlights the context-dependent nature of its function. Exercise can cause a slight, acute increase in IL-6 in the body, which has direct anti-inflammatory benefits (15, 89). In contrast, more intense or prolonged bouts of exercise can lead to a greater production of inflammatory cytokines (90).

A deeper understanding of IL-6’s pleiotropic role reveals that its function is not simply to suppress inflammation but to actively steer the immune response toward resolution (91). For instance, IL-6 has been shown to enhance the capacity of human macrophages to phagocytose apoptotic cells, a critical step in inflammation resolution (92). Furthermore, by boosting the release of IL-4 and IL-10, IL-6 encourages an anti-inflammatory cytokine profile (92). It also favors the alternative immunoregulatory/M2-like macrophage activation state, which is associated with tissue repair and anti-inflammatory functions, while attenuating pro-inflammatory/M1-like phenotype (87). This positions exercise-induced IL-6 as a crucial link between physical activity and the active resolution of inflammation.

### Beyond IL-6: additional pathways and mediators

While IL-6 is a prominent mediator, exercise-induced immune modulation involves a broader network of cytokines, neuroendocrine factors, and neural signals that interact synergistically (45). For instance, other myokines such as IL-15 and IL-7 are released during muscle contraction and contribute to immune regulation. IL-15 promotes the survival and proliferation of natural killer (NK) cells and T lymphocytes, enhancing immunosurveillance, while IL-7 supports lymphocyte homeostasis and may aid in anti-inflammatory responses (46). Neuroendocrine mediators, including catecholamines (e.g., adrenaline and noradrenaline) released via sympathetic activation, modulate immune cells through β-adrenergic receptors, influencing cytokine production and shifting metabolism toward anti-inflammatory states (45, 93). Glucocorticoids like cortisol, elevated during acute exercise, exert anti-inflammatory effects by suppressing pro-inflammatory cytokines but can become immunosuppressive if chronically elevated (45). Additionally, neural inputs, such as vagus nerve signaling in the cholinergic anti-inflammatory pathway, provide rapid modulation of inflammation by inhibiting cytokine release from macrophages (94). This multi-factorial system highlights the limitations of an IL-6-centered framework: overemphasizing IL-6 may overlook synergistic or compensatory roles of these mediators, potentially leading to an incomplete understanding of exercise’s immunomodulatory effects. Future studies should integrate these pathways to better elucidate their interactions (45).

### Myokines and immunometabolism

**Figure 2:**
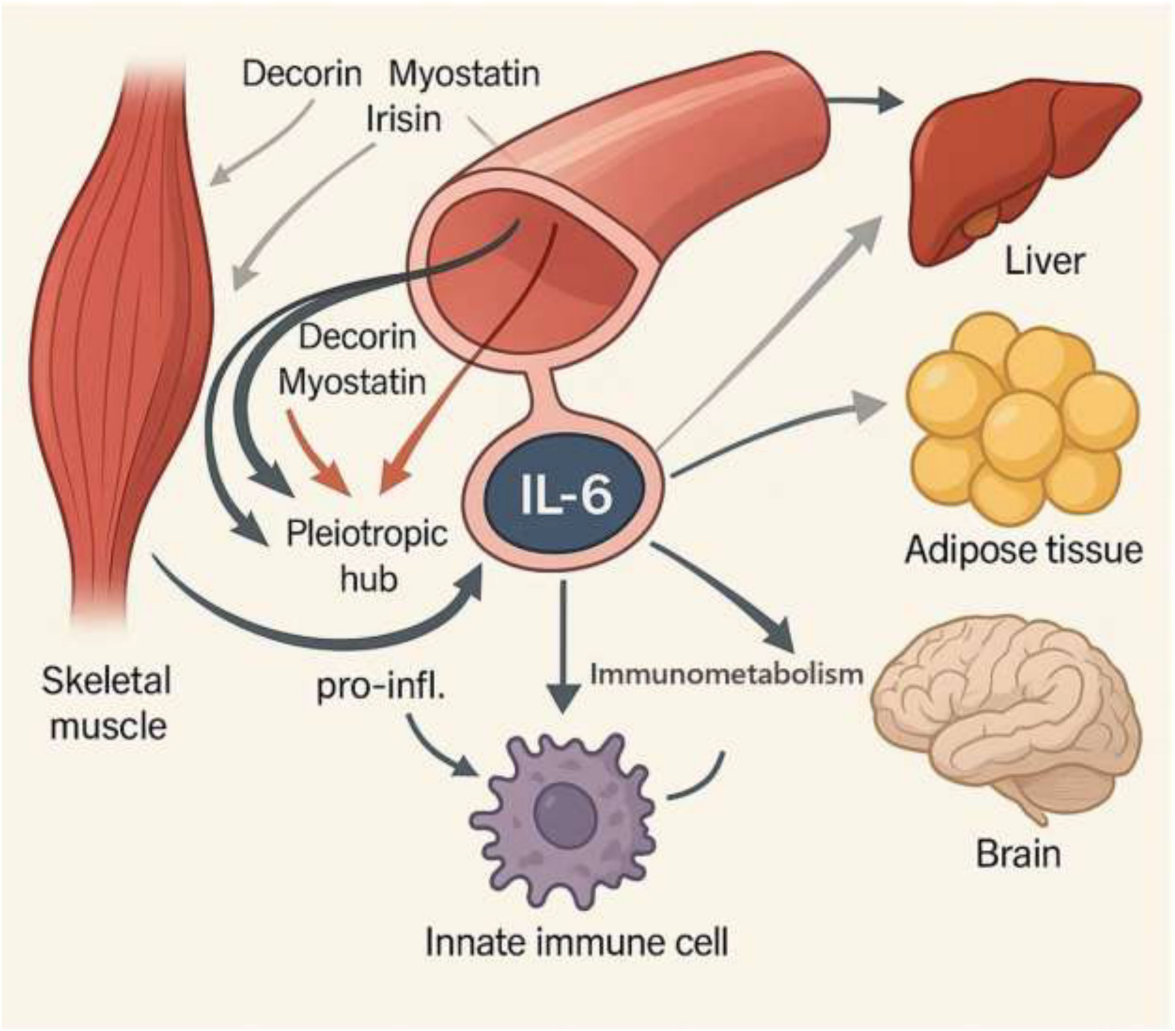
Myokines: The endocrine arm of exercise. Myokines, which are systemic messengers that target organs like the liver, adipose tissue, and brain, are released when skeletal muscle contraction occurs. These include IL-6, Decorin, Myostatin, and Irisin. IL-6 serves as a pleiotropic hub, exerting dual effects: acute, exercise-induced IL-6 promotes anti-inflammatory resolution by enhancing macrophage phagocytosis, inducing IL-4/IL-10 secretion, and driving M2 macrophage polarization; prolonged or excessive IL-6 contributes to pro-inflammatory signaling. Myokines also regulate immunometabolism, shifting immune cells from oxidative phosphorylation toward glycolysis and supporting fatty acid oxidation and lipolysis. This mechanistic axis links exercise to immune regulation, inflammation resolution, and metabolic adaptation.

Immune cell metabolic reprogramming is inextricably tied to the signaling cascade that myokines start (95, 96). Trained immunity, as previously discussed, requires a metabolic shift to meet the high energy demands of cellular activation (1). Exercise, by its very nature, is a powerful modulator of systemic metabolism (25). The causal link between these two phenomena is the myokine-immunometabolism axis (97). Exercise-induced myokines act as systemic signals that directly influence the metabolic programs of innate immune cells (78). This effect may help to explain how some metabolic changes (such as a switch from OXPHOS to glycolysis) that are characteristic of trained immunity can be induced by a systemic stimulus like exercise (67). These changes align with the broader metabolic adaptations in trained immunity, including TCA cycle flux and lipid metabolism, as modulated by exercise (54). Myokines like IL-6, which can stimulate fatty acid oxidation and lipolysis, provide the necessary energetic resources for this metabolic transition (87). This establishes a tangible, mechanistic pathway by which exercise “trains” the innate immune system (**Figure 2**, **Table 1**).

**Table 1:** Important myokines induced by exercise and their immunomodulatory impacts.

Myokine	Primary stimulus	Target cell/organ	Effect on immunity
Interleukin-6 (IL-6)	Muscle contraction (aerobic/resistance)	Macrophages, Adipose Tissue, Liver	Enhances macrophage phagocytosis, promotes M2 anti-inflammatory phenotype, signals for inflammation resolution
Decorin	Muscle contraction	Adipose Tissue, Liver	Anti-inflammatory and anti-fibrotic effects, reduces hepatic inflammation
Myostatin	Muscle contraction	Adipose Tissue, Liver	Lower plasma levels reduce pro-inflammatory factors like TNFα
Irisin	Muscle contraction	Adipose Tissue, Liver	May induce anti-inflammatory response, although high levels are also linked to inflammation
Brain-Derived Neurotrophic Factor (BDNF)	Muscle contraction	Neurons, Immune cells	Promotes neuronal survival, modulates immune cell function
Leukemia Inhibitory Factor (LIF)	Muscle contraction	Immune cells	Promotes immune cell proliferation and differentiation
Interleukin-15 (IL-15)	Muscle contraction	NK cells, T lymphocytes	Promotes survival and proliferation of NK and T cells, enhances immunosurveillance
Interleukin-7 (IL-7)	Muscle contraction	Lymphocytes	Supports lymphocyte homeostasis and anti-inflammatory responses

## Resolving inflammation: the final phase of the immune response

In order to restore tissue homeostasis and avoid chronic inflammatory illness, inflammation resolution is an active, coordinated process (9, 98). This section will detail the key molecular and cellular players in this process and demonstrate how exercise directly contributes to its efficiency.

### The pillars of inflammation resolution

Following the initial acute inflammatory response, a programmed resolution phase, known as catabasis, is initiated (9, 99). This process is characterized by a set of “five pillars,” which include the active elimination of bacteria, debris, and dead cells; vascular integrity restoration; tissue regeneration; fever remission; and inflammatory pain decrease (9). Chronic inflammatory disorders are largely caused by the failure of this active mechanism (100).

### Specialized pro-resolving mediators

A cornerstone of inflammation resolution is a class of signaling molecules known as SPMs (9). Lipid mediator class-switching is the process by which these are enzymatically produced from necessary fatty acids (9). Key families of SPMs include lipoxins, resolvins (e.g., Resolvin D1), protectins, and maresins (9). SPMs differ from immunosuppressive compounds in that they actively support host defense, the removal of pathogens and cellular detritus, and inflammation reduction (9). Regular physical activity has been shown to potentially accelerate and strengthen the pro-resolving lipid mediator response following acute exercise stress (101). Furthermore, Exercise increases RvD1 levels and stimulates macrophage phagocytosis, a crucial pro-resolving mechanism, according to study (28) (**Table 2**).

**Table 2:** SPMs and their mechanisms.

SPM family	Precursor fatty acid	Key pro-resolving actions	Link to exercise
Resolvins (D/E-series)	Omega-3 PUFAs (DHA/EPA)	Promotes clearance of pathogens and apoptotic cells, inhibits neutrophil infiltration, stimulates anti-inflammatory cytokine production	Exercise enhances Resolvin D1 levels and strengthens the response to acute stress (28)
Lipoxins	Omega-6 PUFAs (Arachidonic Acid)	Inhibits neutrophil recruitment and promotes their non-inflammatory apoptosis, suppresses pro-inflammatory signaling	Exercise may influence the class-switching from pro-inflammatory to pro-resolving lipid mediators (9)
Maresins	Omega-3 PUFAs (DHA)	Augment macrophage phagocytosis and microbial killing, promote tissue repair	Potential link, but specific research on maresins and exercise is an area for future study
Protectins	Omega-3 PUFAs (DHA)	Neuroprotective, anti-inflammatory, and promotes efferocytosis	Potential link, but specific research on protectins and exercise is an area for future study

### Efferocytosis: a critical cellular mechanism

Efferocytosis, a carefully controlled biological process, occurs when macrophages quickly eliminate apoptotic cells (77, 102, 103). This procedure is essential for stopping necrotic cells from releasing pro-inflammatory DAMPs, which can cause sterile inflammation and worsen chronic illness (77, 104). The timely clearance of apoptotic cells is, in itself, a powerful pro-resolving signal (77). The degradation of these cells provides macrophages with key metabolites, like fatty acids, amino acids, and cholesterol, which causes the macrophage to change into a pro-resolving and anti-inflammatory phenotype (77).

An intricate nexus exists between exercise, myokine signaling, and efferocytosis. Exercise-induced IL-6 increases human macrophages’ capacity to phagocytose apoptotic cells (92, 105). This creates a clear, multi-layered causal pathway: exercise triggers the systemic release of myokines and SPMs, which in turn enhance efferocytosis and fuel the pro-resolving macrophage phenotype (106). This mechanism elegantly demonstrates how physical activity actively promotes the restoration of tissue homeostasis, providing a robust defense against chronic inflammatory conditions (92).

## Unifying the concepts: a model of exercise-induced immune modulation

The complex relationship between exercise, trained immunity, and inflammation resolution can be synthesized into a unified model that connects molecular, cellular, and systemic effects (107). This model resolves the paradox of exercise’s dual effects on immunity and provides a comprehensive framework for its physiological benefits (107, 108).

Physical activity, particularly moderate, regular exercise, acts as a controlled physiological stressor (15, 109). Skeletal muscle’s endocrine activity is triggered by this stimulus, which causes the systemic release of several myokines, the main one being IL-6 (25). This systemic myokine signaling, in turn, influences the metabolism of innate immune cells (78). This metabolic shift provides the necessary energetic and material resources to fuel the epigenetic changes at key inflammatory gene promoters and enhancers that define trained immunity (1). This long-term reprogramming leads to a persistent state of enhanced immunosurveillance and heightened responsiveness to pathogens (15).

Simultaneously, myokines like IL-6 directly increase efferocytosis, and exercise stimulates the synthesis of pro-resolving lipid mediators like SPMs (28, 110). In addition to preventing the development of sterile inflammation, this active removal of apoptotic cells also strengthens the pro-resolving macrophage character, ensuring the efficient and timely resolution of inflammation (77).

This model provides a compelling explanation for the J-shaped curve of exercise and illness risk. Moderate, regular exercise is associated with inducing a beneficial, transient inflammatory state that is followed by a robust, myokine- and SPM-driven resolution phase (15). This repeated, controlled “training” of the immune system leads to chronic adaptations (107). In contrast, heavy, chronic training may induce an inflammatory state so profound or persistent that the resolution mechanisms are overwhelmed, leading to the observed transient immunosuppression and increased risk of minor infections (111) (**Table 3**). The key is the delicate balance between the initial pro-inflammatory stimulus and the subsequent pro-resolving response.

**Table 3:** Exercise's acute and chronic effects on innate immune cells.

Cell type	Acute exercise (<60 min, moderate)	Acute prolonged strenuous exercise (>90 min)	Chronic regular exercise
Neutrophils	Increased recirculation, Enhanced phagocytosis	Transient decrease, Depressed phagocytosis	Enhanced immune regulation
NK Cells	Increased recirculation and cytotoxicity	Significant decrease in circulating numbers	May be elevated at rest in some athletes, improved function
Monocytes	Increased recirculation	Significant decrease in circulating numbers, reduced function	Decreased systemic inflammation, enhanced function
Macrophages	Enhanced anti-pathogen activity	Reduced antigen presentation	Enhanced efferocytosis, anti-inflammatory phenotype

## Knowledge gaps, conflicting evidence, and future directions

Exercise immunology has made great strides, yet there are still debates and unresolved issues in the field. A critical appraisal of the existing literature is essential for advancing our understanding and translating these findings into clinical practice.

### The “open window” debate and its methodological limitations

The “open window” hypothesis, which posits a temporary period of immunosuppression after strenuous exercise, is a cornerstone of exercise immunology (19, 112). However, some contemporary evidence and critical reviews argue that there is limited reliable data to support this claim (16). A major contributing factor to this debate and the often-conflicting results in the literature is the reliance on traditional *ex vivo* immune markers. Clinical outcomes including the likelihood of upper respiratory tract infections are not necessarily correlated with these markers, such as variations in circulating cell counts or cytokine levels in blood tests (113). A temporary change in a single immune variable in the blood does not necessarily equate to a compromised host defense, due to the inherent redundancy and compensatory mechanisms of the immune system (113, 114).

Future research must move beyond these correlational studies and adopt more rigorous methodologies (115). The use of controlled *in vivo* infection challenge models is necessary to establish a direct causal link between exercise-induced changes and host defense against pathogens (113). Additionally, Research should take a multifaceted approach, taking into consideration additional variables that affect immunological health, such sleep, as exposure to pathogens, nutrition, and psychological stress (112).

### Specific gaps in our understanding

A comprehensive understanding of the exercise-immunity nexus requires addressing several key knowledge gaps:

- It is still unclear the precise signaling pathways connect exercise-induced myokines to the metabolic and epigenetic remodeling of HSCs (1, 116). Direct evidence linking exercise to epigenetic shaping of the immune cell landscape is emerging but remains limited; while overlaps with trained immunity exist, many studies are associative, extrapolated from non-exercise models.
- The full spectrum of myokines involved in immune regulation is yet to be fully characterized, with most research focusing on a limited number of molecules like IL-6 (46).
- There is clear absence enough research of on the infection risk and immune adaptations in predominantly strength-based athletes, despite the similar immunological changes observed in aerobic and resistance exercise (113, 117, 118).
- The precise upstream signals that trigger the release of exercise-induced molecules like cell-free DNA (cfDNA) are not the major inflammatory cytokines, suggesting other, yet-to-be-discovered mechanisms (119).
- Much of the evidence linking exercise to trained immunity and HSC reprogramming is indirect or associative, often extrapolated from non-exercise models like BCG or β-glucan; direct causal studies in exercise contexts are needed (45).

## Future directions

To fully elucidate the complex interplay between exercise, trained immunity, and inflammation resolution, we advocate for a multi-omics approach that integrates data from the genome, epigenome, transcriptome, proteome, metabolome, and microbiome (1, 120). This systems biology approach will allow for a holistic understanding of the intricate networks that regulate exercise-induced immune modulation (1).

Furthermore, clinical research should employ rigorous methodology, including controlled infection challenge models, to move beyond correlational *ex vivo* data and establish direct causal links between exercise and host defense (113). Future research should also look into how various exercise styles (such as resistance versus aerobic) affect the myokine profile and how that affects trained immunity and the resolution of inflammation (121). Ultimately, gaining a comprehensive understanding of these pathways is essential for creating innovative, focused treatment strategies to fight chronic inflammatory disorders and infectious diseases (58).

## Conclusion

Exercise emerges as a powerful physiological modulator of immune function by bridging two rapidly evolving concepts in immunology: trained immunity and inflammation resolution. Through coordinated epigenetic reprogramming and metabolic rewiring, exercise imprints long-lasting functional adaptations on innate immune cells, enhancing their capacity for immunosurveillance while simultaneously fostering a pro-resolving phenotype. As molecular conduits, central mediators like myokines (particularly IL-6) and SPMs connect tissue homeostasis and systemic immune control to skeletal muscle function. Together, these mechanisms explain how regular physical activity not only strengthens resistance against pathogens but also mitigates chronic inflammation, a common denominator in aging and numerous non-communicable diseases. While significant progress has been made, critical knowledge gaps remain, particularly in defining the temporal dynamics of exercise-induced innate memory, various exercise techniques’ dosage and response, and the integration of multi-omics approaches to unravel this complexity *in vivo*. Future research should aim to translate these mechanistic insights into targeted exercise-mimetic therapies or personalized training prescriptions, harnessing the dual potential of exercise to train immunity and resolve inflammation. Ultimately, this concept presents exercise as a biologically based therapeutic approach for fostering resilience, avoiding illness, and prolonging health span rather than just as a lifestyle intervention.
